# Supplementary material for: A-Type Natriuretic Peptide Alters the Impact of Azithromycin on Planktonic Culture and on (Monospecies and Binary) Biofilms of Skin Bacteria Kytococcus schroeteri and Staphylococcus aureus
Source: Microorganisms. 2023 Dec 12;11(12):2965. doi: 10.3390/microorganisms11122965 (PMC10746058; doi:10.3390/microorganisms11122965)
Supplement: Supplementary file 1 [file microorganisms-11-02965-s001.zip › Supplementary Table S1.pdf]

Supplementary Table S1. Growth kinetics parameters of monospecies and binary cultures of *K. schroeteri* and *S. aureus* in different model systems.

Parameter			Maximal OD_{540}	Specific growth rate, h^{-1}	Genetation time, h	Linear portion of the growth curve, h
Model system	Microorganism	Sample				
With forced initial adhesion	*K. schroteri*	Control	1.972	0.186	4.502	1.167
		Az 0.001 µg/mL	1.847	0.188	4.969	1.167
		Az 4 µg/mL	1.27	0.119	21.207	2.167
		Az 0.001 µg/mL + ANP	1.825	0.187	4.996	1.333
		Az 4 µg/mL + ANP	1.269	0.133	20.205	2.000
	S. aureus	Control	1.348	0.267	2.600	1.667
		Az 0.001 µg/mL	1.156	0.214	2.668	1.417
		Az 4 µg/mL	1.011	0.098	8.386	1.417
		Az 0.001 µg/mL + ANP	1.498	0.292	2.382	1.500
		Az 4 µg/mL + ANP	1.276	0.134	7.273	1.833
	Binary	Control	1.596	0.244	2.866	1.500
		Az 0.001 µg/mL	1.551	0.198	3.621	1.500
		Az 4	1.718	0.110	6.863	1.833

		µg/mL				
		Az 0.001 µg/mL + ANP	1.560	0.213	3.287	1.500
		Az 4 µg/mL + ANP	1.698	0.137	5.294	2.000
Without forced initial adhesion	*K. schroteri*	Control	1.703	0.210	3.743	1.500
		Az 0.001 µg/mL	1.616	0.278	1.647	1.083
		Az 4 µg/mL	0.975	0.210	3.609	1.583
		Az 0.001 µg/mL + ANP	1.661	0.260	2.895	1.500
		Az 4 µg/mL + ANP	1.090	0.228	3.310	1.750
	S. aureus	Control	1.277	0.239	2.980	1.750
		Az 0.001 µg/mL	1.042	0.194	3.164	1.417
		Az 4 µg/mL	0.923	0.047	15.252	2.250
		Az 0.001 µg/mL + ANP	1.237	0.239	3.112	2.000
		Az 4 µg/mL + ANP	1.027	0.058	12.026	1.583
	Binary	Control	1.265	0.227	3.156	1.917
		Az 0.001 µg/mL	1.269	0.212	3.362	1.500
		Az 4	1.218	0.132	6.734	2.000

	µg/mL				
	Az 0.001 µg/mL + ANP	1.264	0.198	3.610	1.833
	Az 4 µg/mL + ANP	1.162	0.13	7.209	2.083
