# Supplementary figures and images for: A-Type Natriuretic Peptide Alters the Impact of Azithromycin on Planktonic Culture and on (Monospecies and Binary) Biofilms of Skin Bacteria Kytococcus schroeteri and Staphylococcus aureus
Source: Microorganisms. 2023 Dec 12;11(12):2965. doi: 10.3390/microorganisms11122965 (PMC10746058; doi:10.3390/microorganisms11122965)

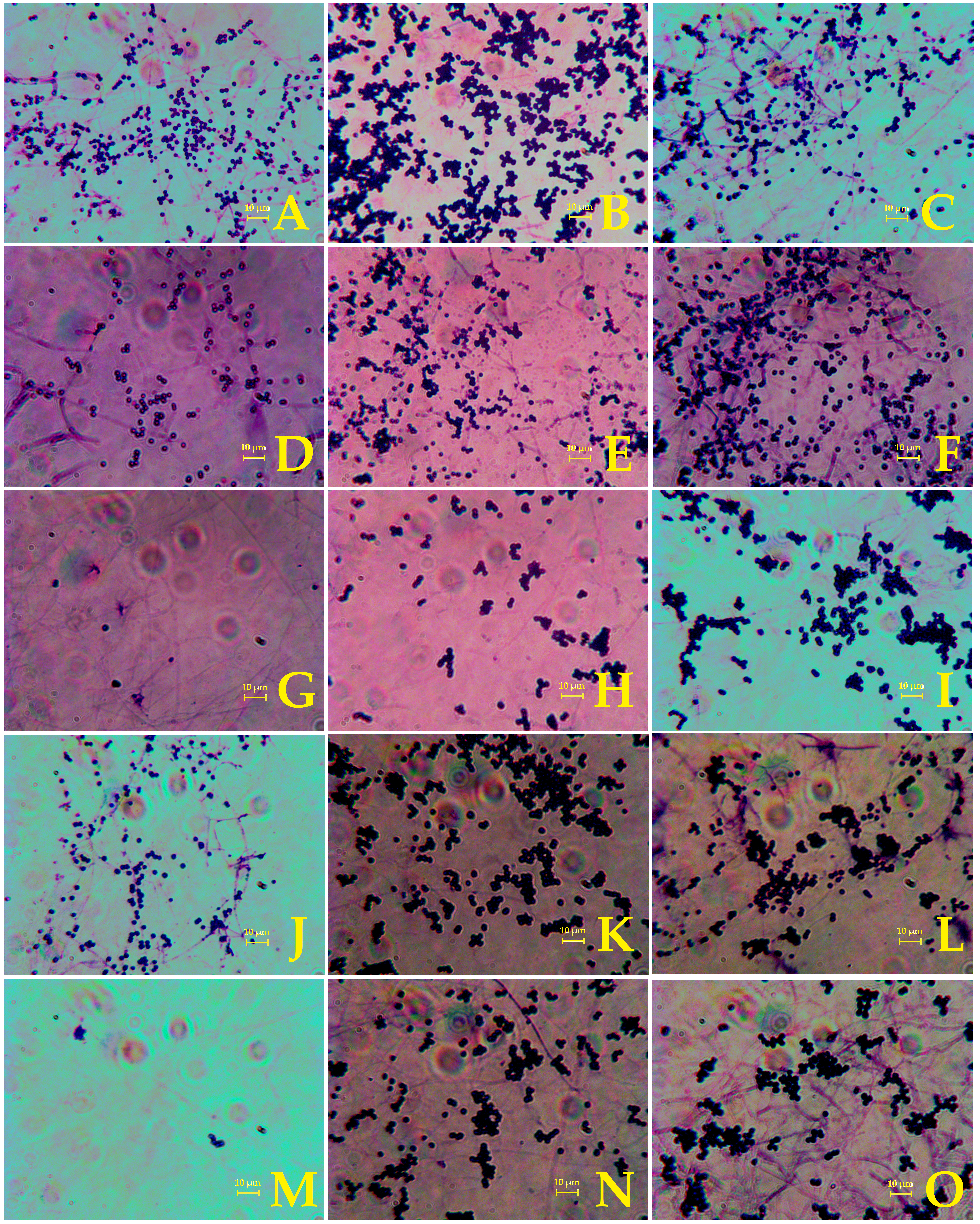

Supplement: Supplementary file 1 [file microorganisms-11-02965-s001.zip › Supplementary Figure S1 plates 24.png]

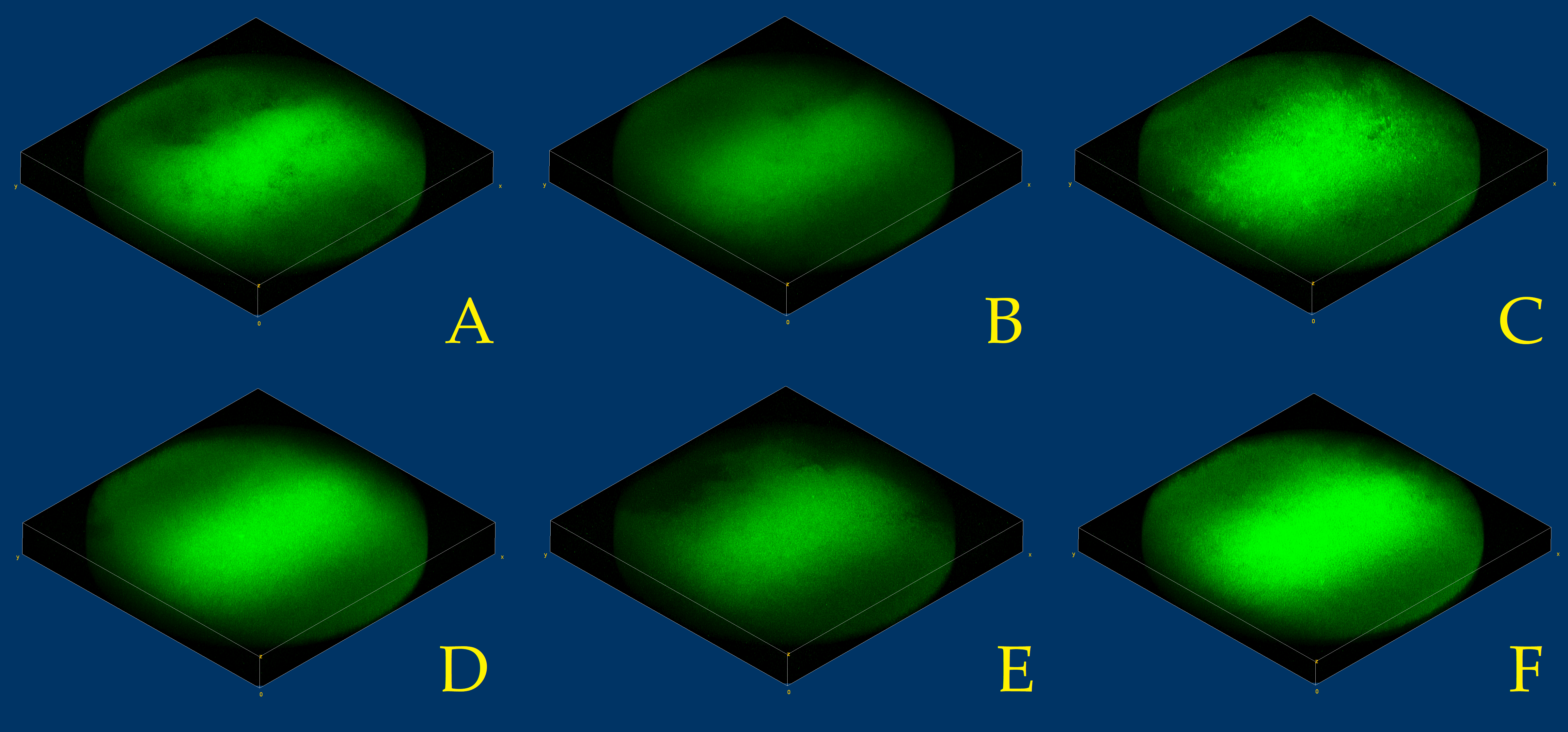

Supplement: Supplementary file 1 [file microorganisms-11-02965-s001.zip › Supplementary Figure S10 kyto 48 CLSM.png]

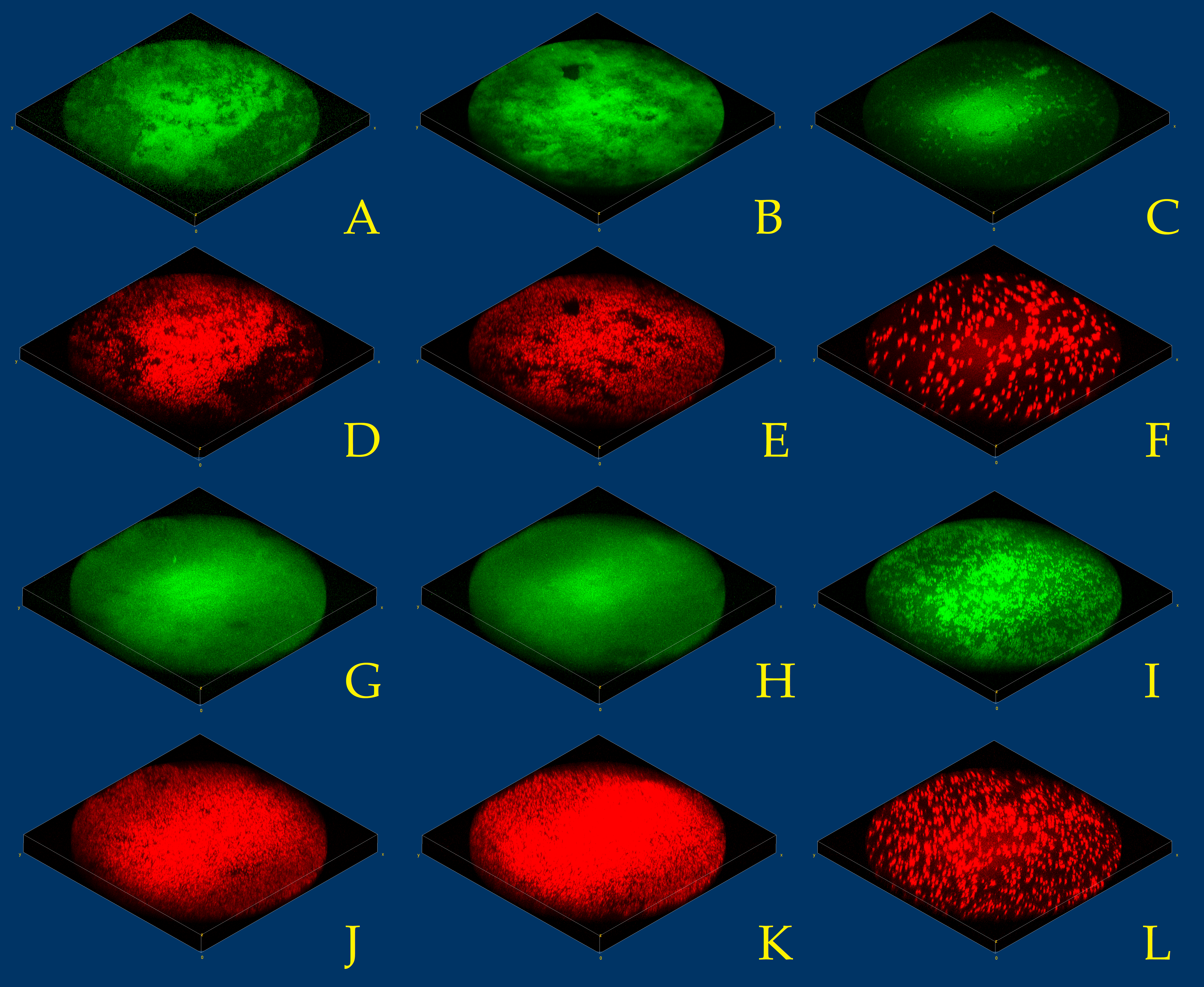

Supplement: Supplementary file 1 [file microorganisms-11-02965-s001.zip › Supplementary Figure S11 staph 48 CLSM.png]

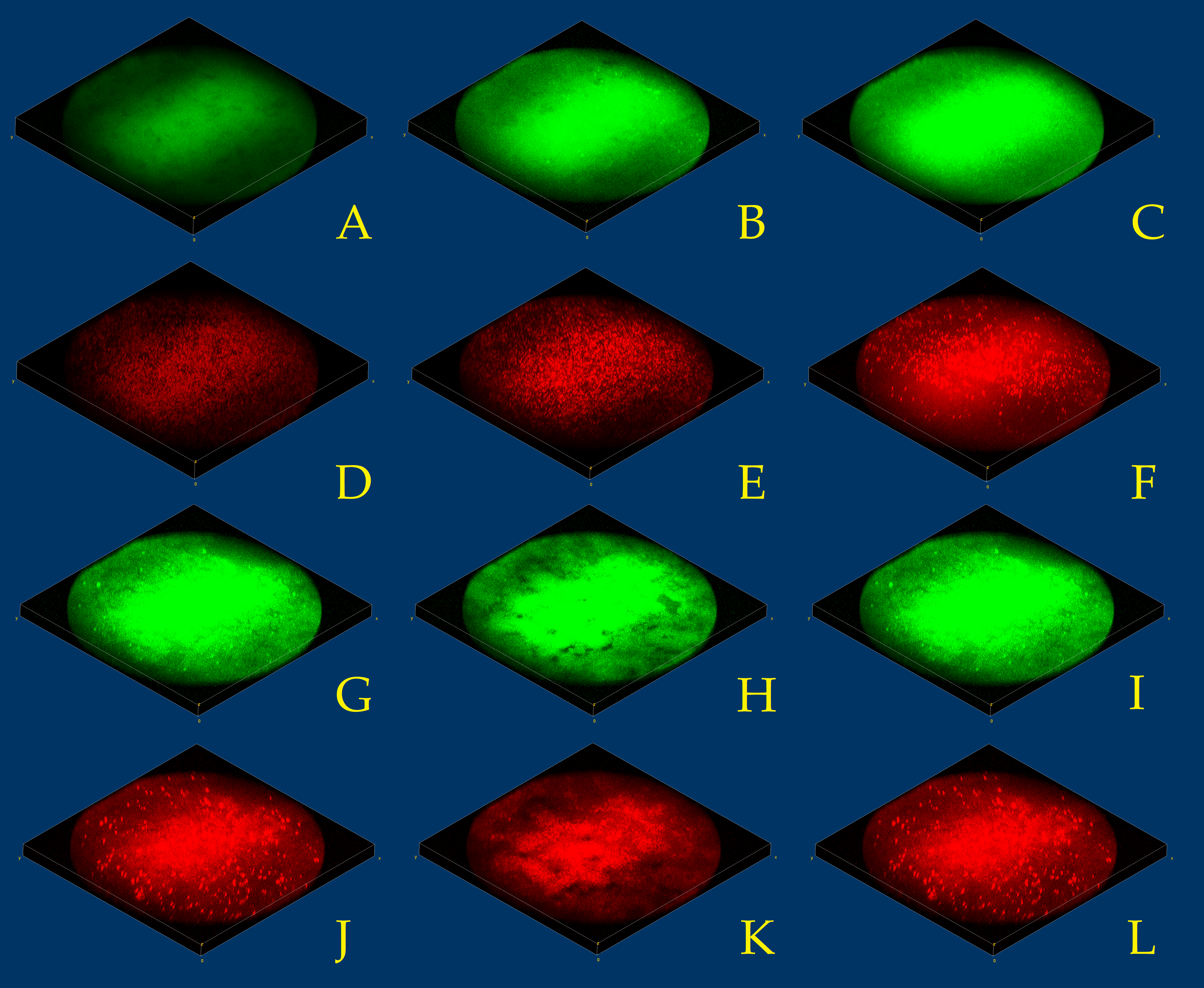

Supplement: Supplementary file 1 [file microorganisms-11-02965-s001.zip › Supplementary Figure S12 binar 48 CLSM.png]

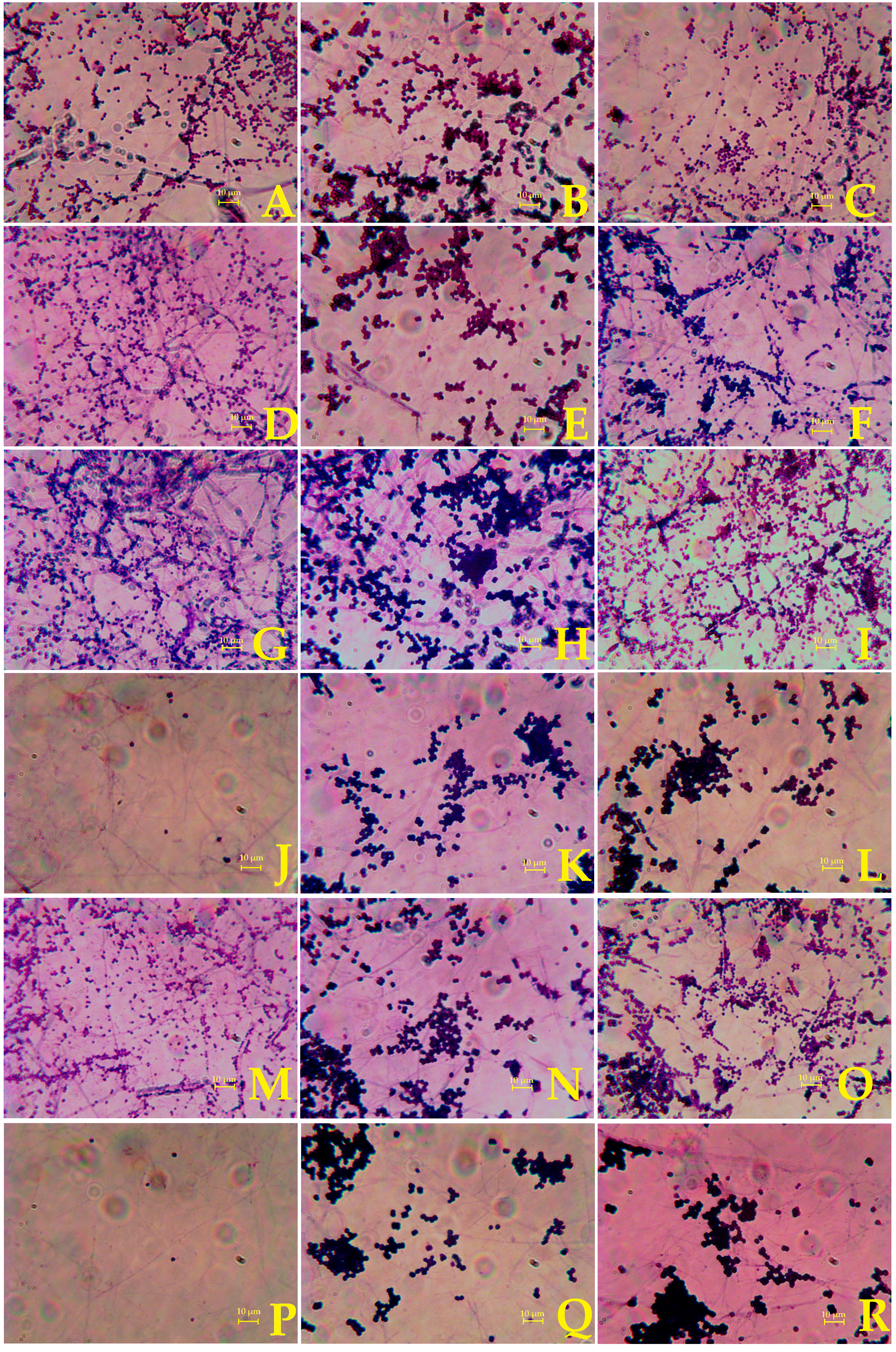

Supplement: Supplementary file 1 [file microorganisms-11-02965-s001.zip › Supplementary Figure S2 plates 48.png]

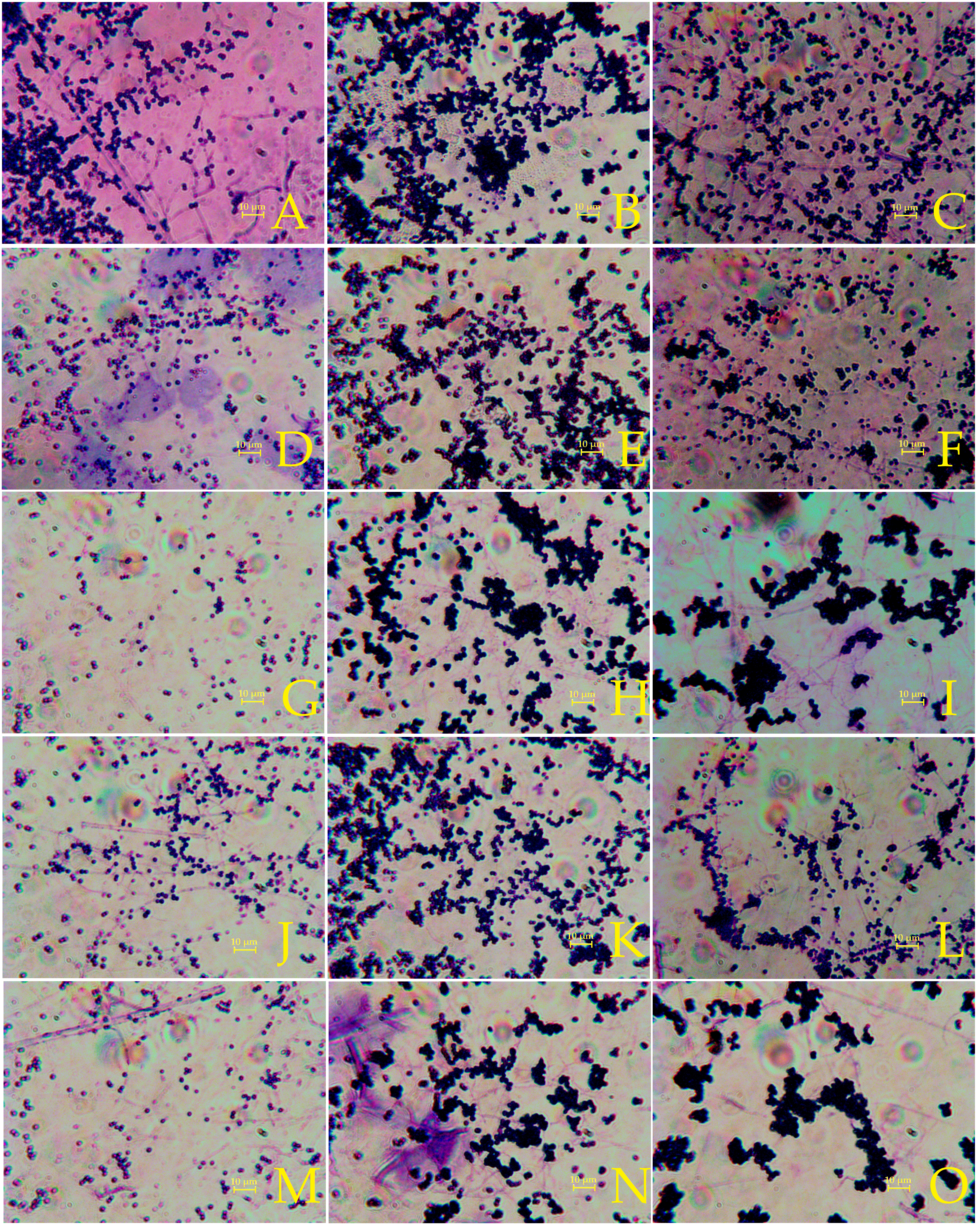

Supplement: Supplementary file 1 [file microorganisms-11-02965-s001.zip › Supplementary Figure S3 plates 72.png]

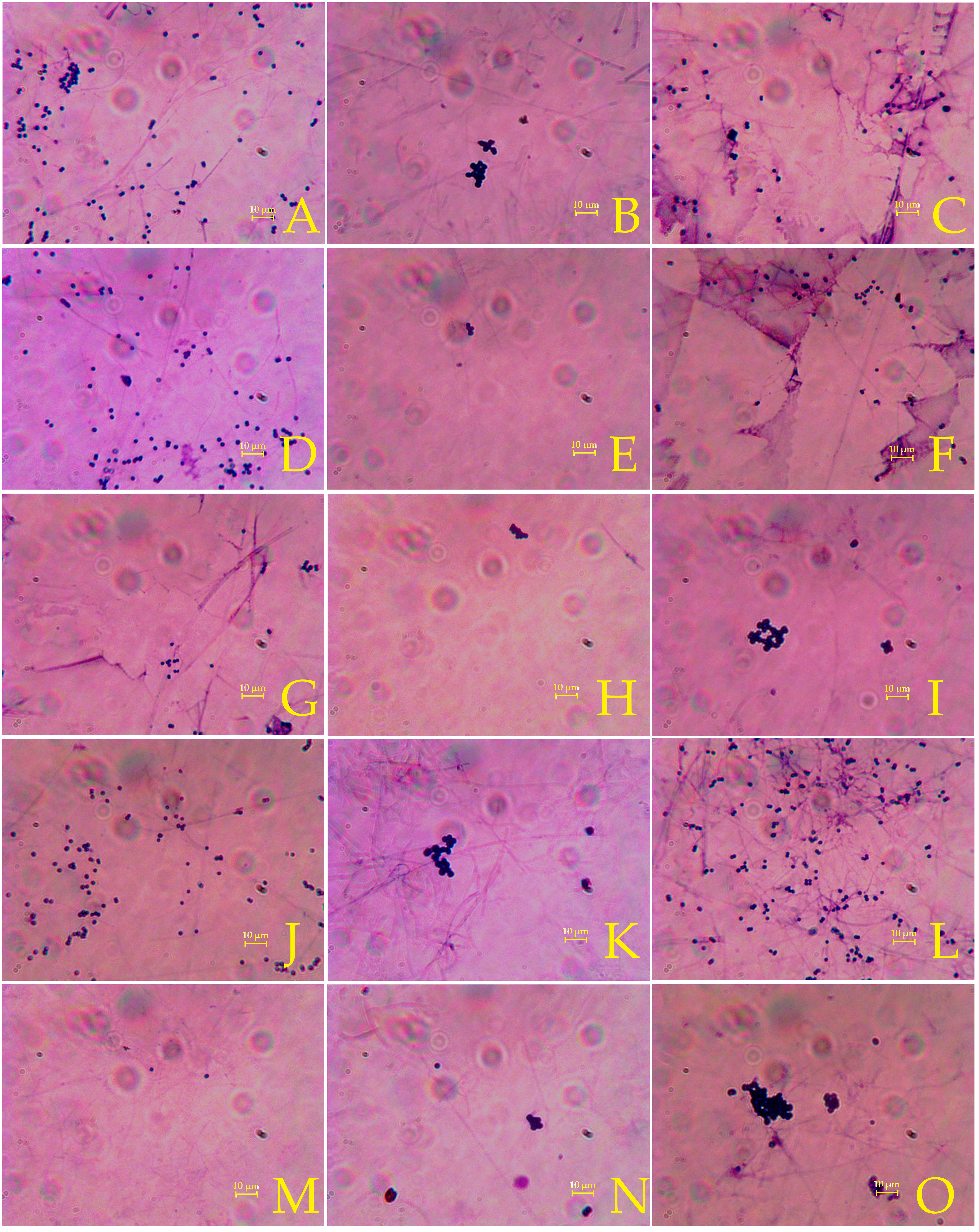

Supplement: Supplementary file 1 [file microorganisms-11-02965-s001.zip › Supplementary Figure S4 tubes 24.png]

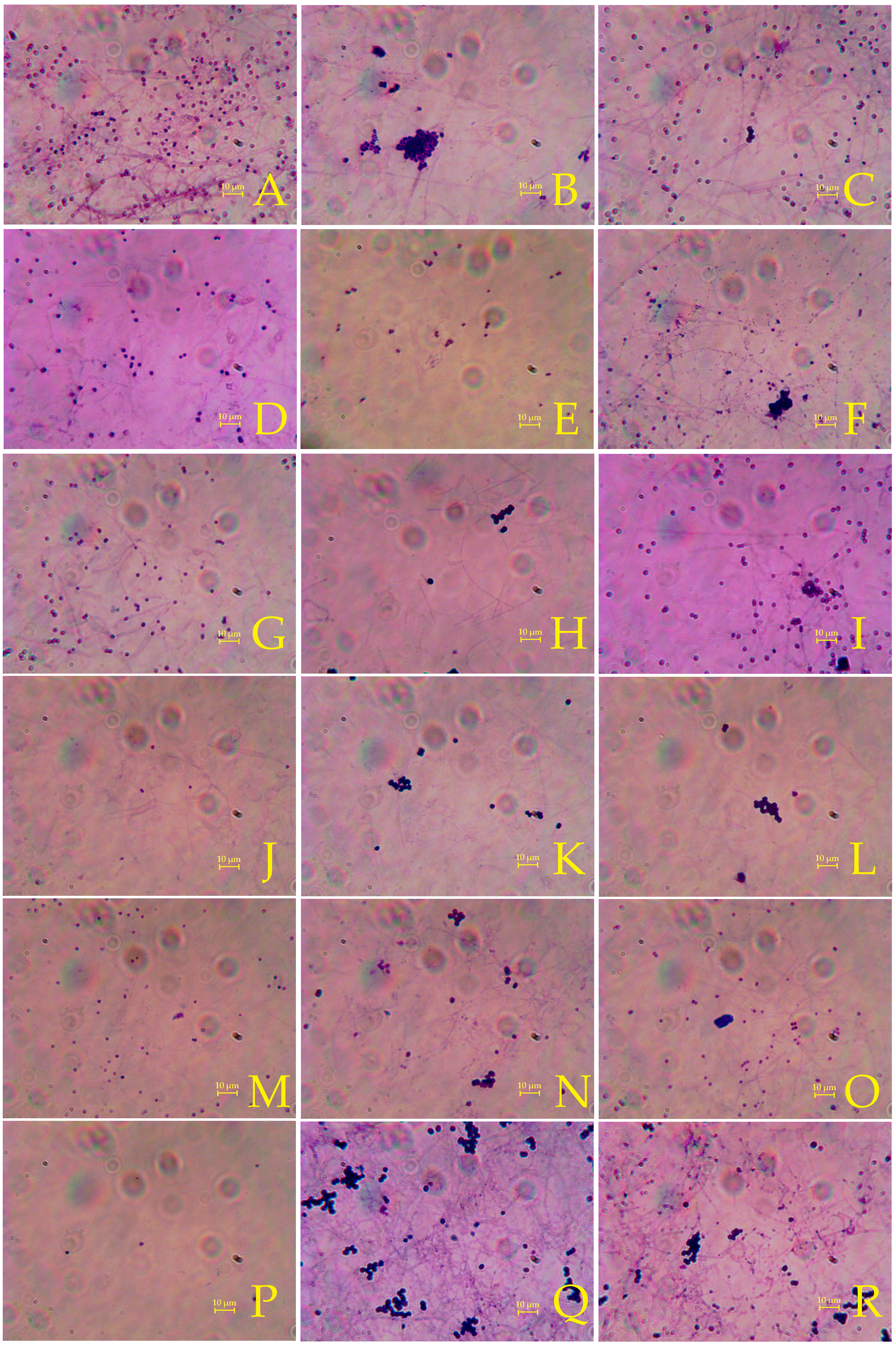

Supplement: Supplementary file 1 [file microorganisms-11-02965-s001.zip › Supplementary Figure S5 tubes 48.png]

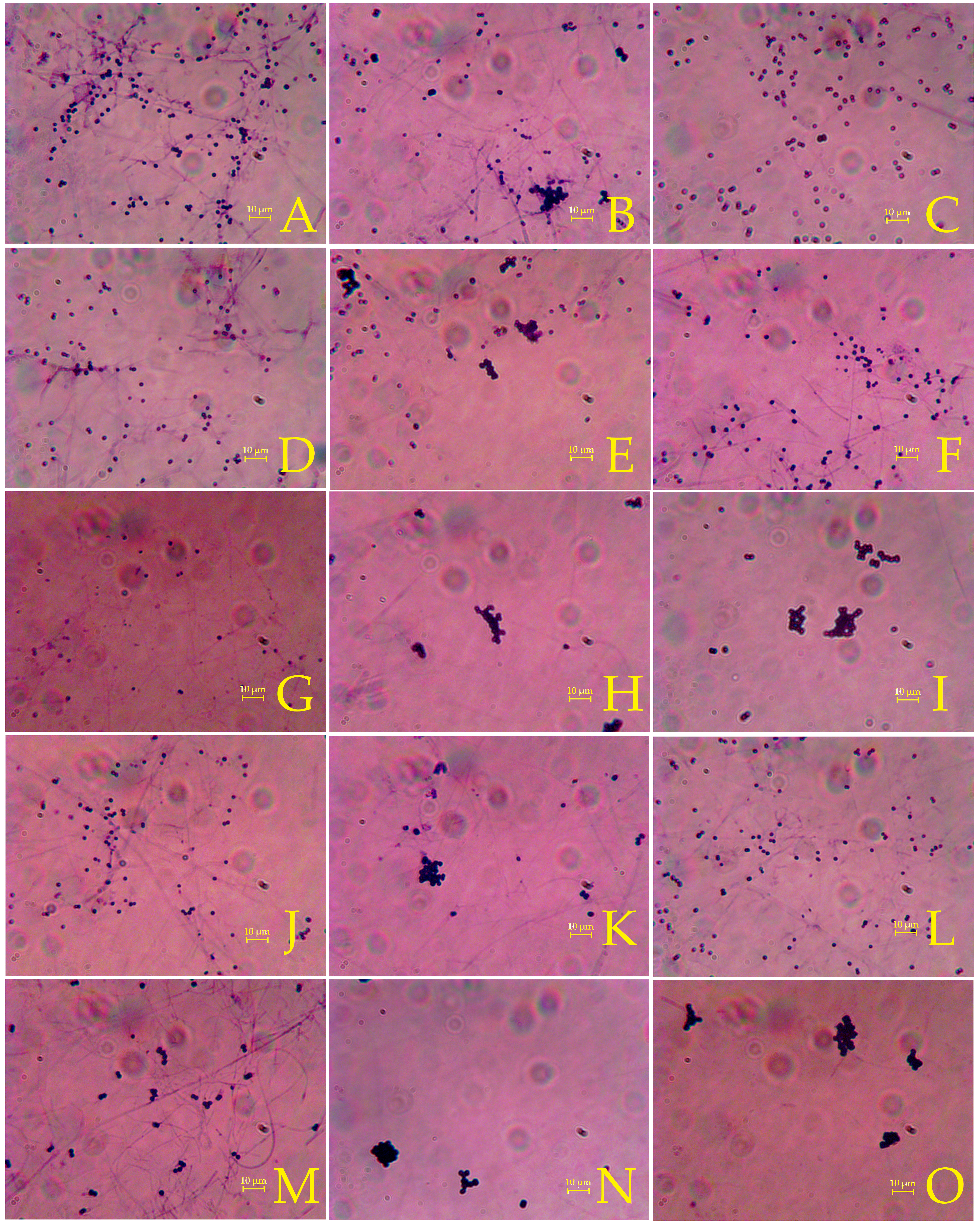

Supplement: Supplementary file 1 [file microorganisms-11-02965-s001.zip › Supplementary Figure S6 tubes 72.png]

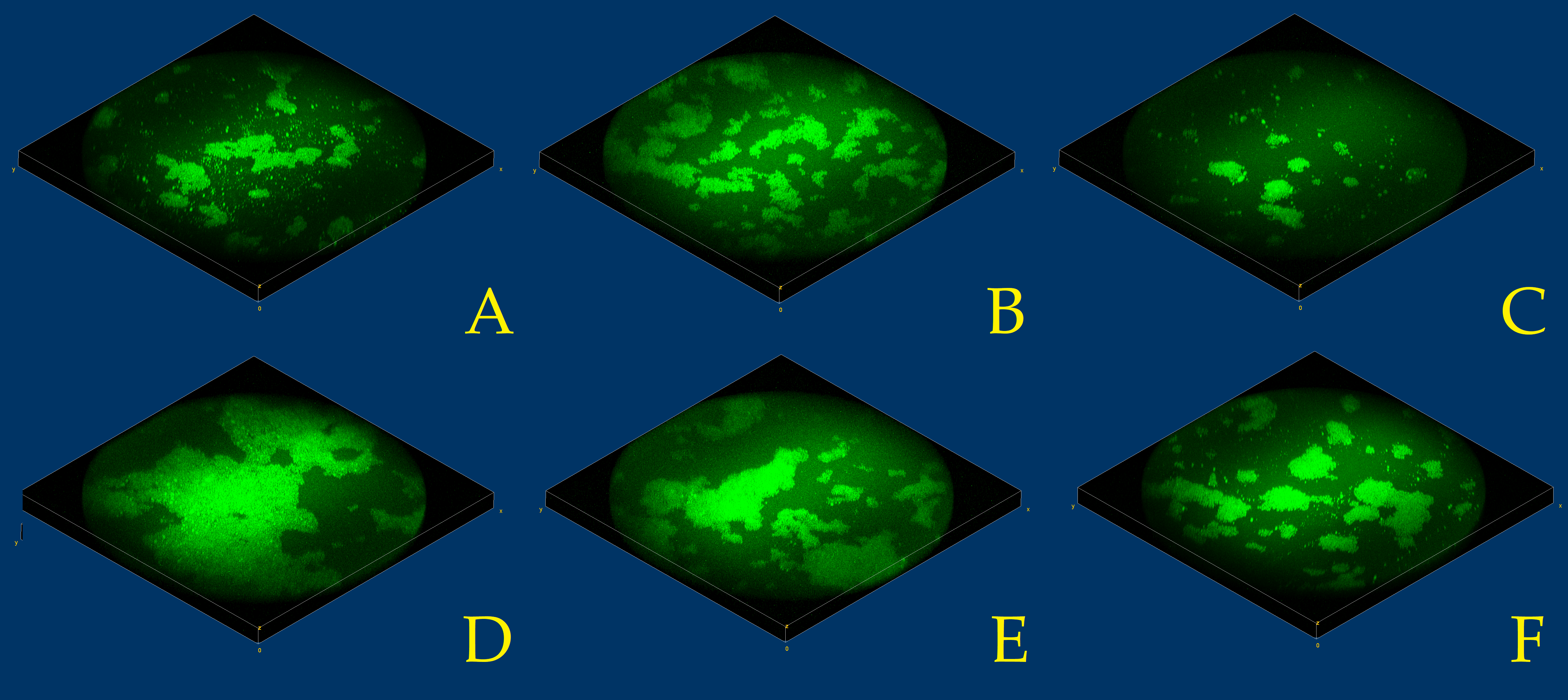

Supplement: Supplementary file 1 [file microorganisms-11-02965-s001.zip › Supplementary Figure S7 kyto 24 CLSM.png]

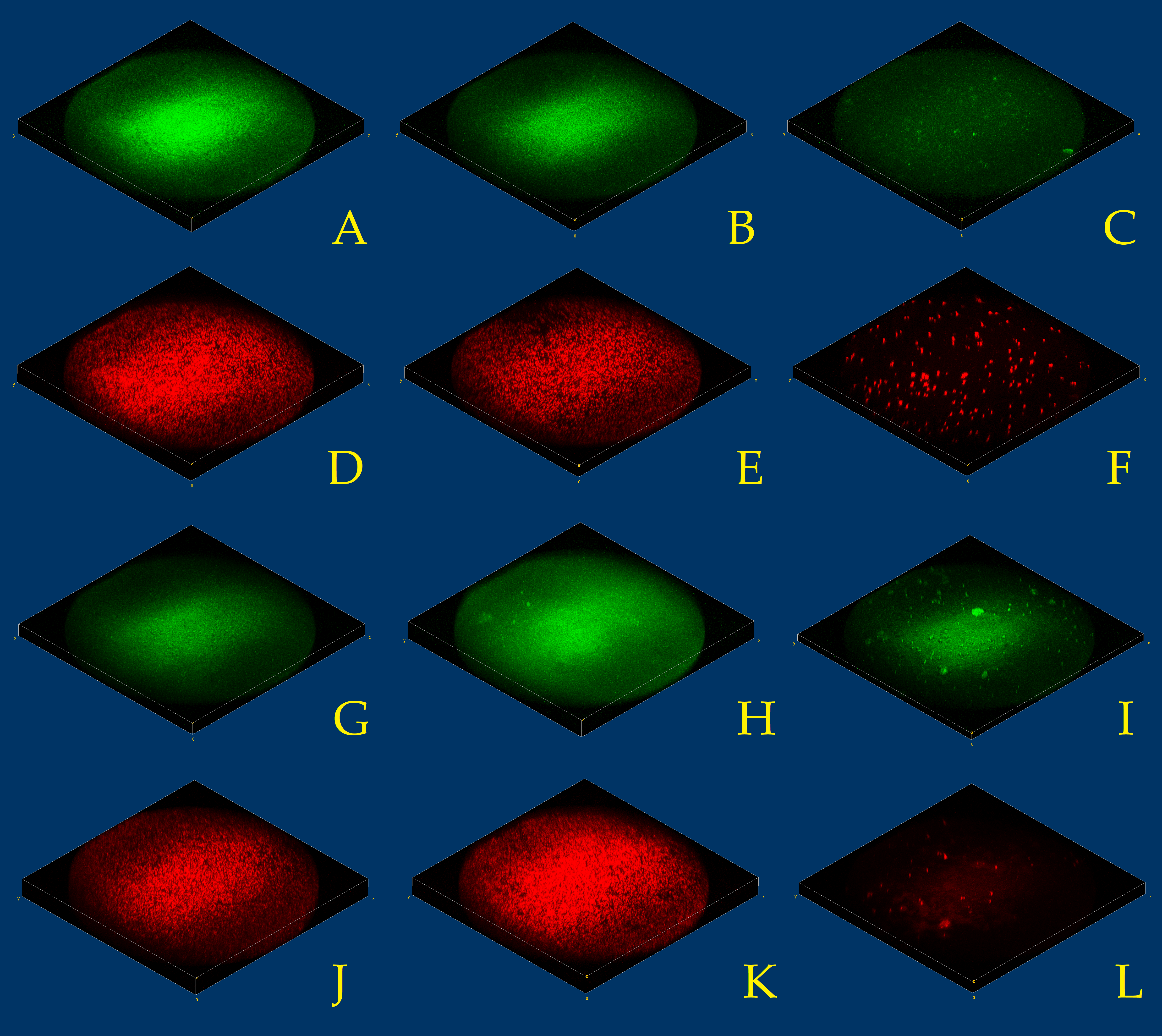

Supplement: Supplementary file 1 [file microorganisms-11-02965-s001.zip › Supplementary Figure S8 staph 24 CLSM.png]

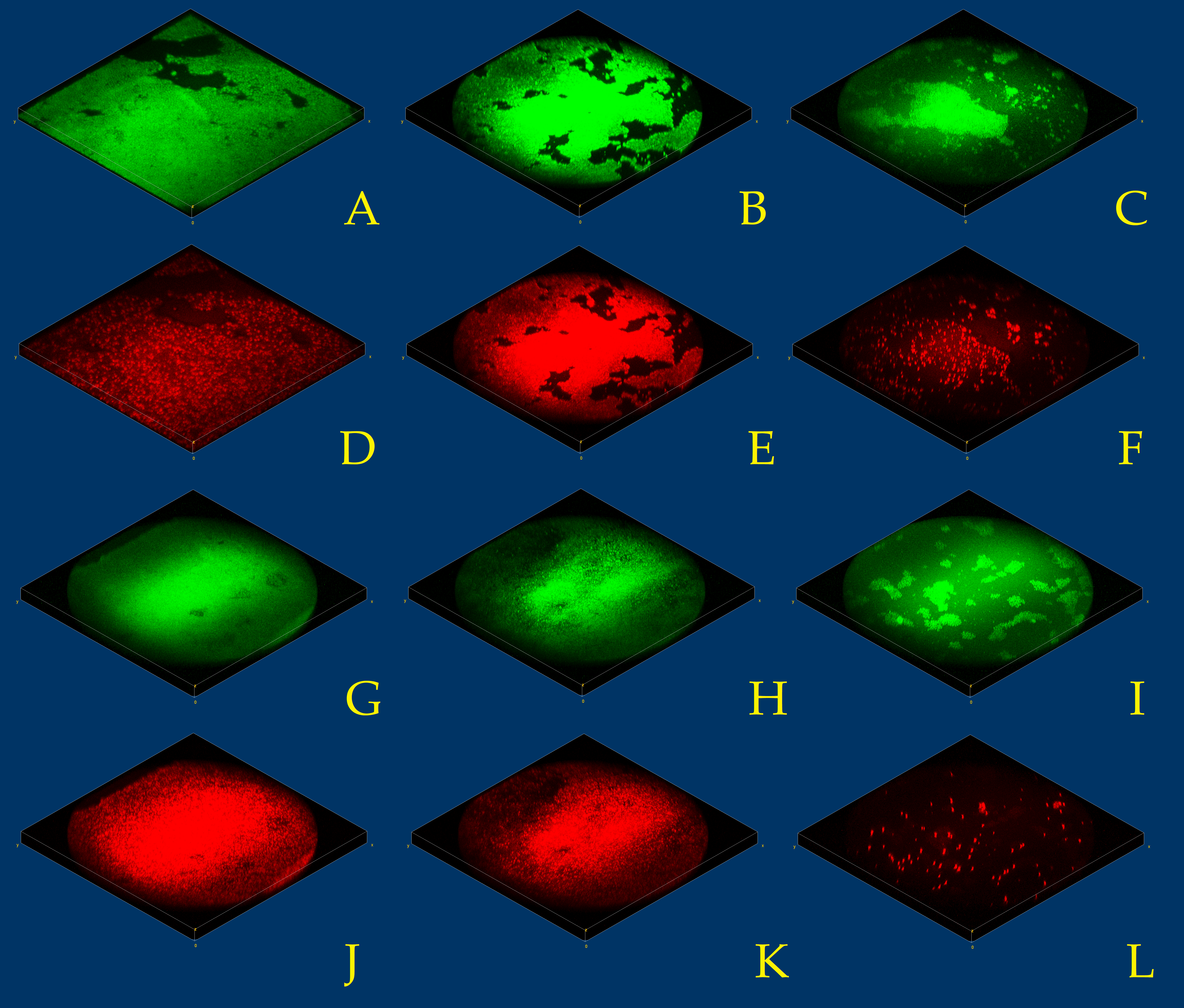

Supplement: Supplementary file 1 [file microorganisms-11-02965-s001.zip › Supplementary Figure S9 binar 24 CLSM.png]
